# Flexural Performance of Encased Pultruded GFRP I-Beam with High Strength Concrete under Static Loading

**DOI:** 10.3390/ma15134519

**Published:** 2022-06-27

**Authors:** Enas M. Mahmood, Abbas A. Allawi, Ayman El-Zohairy

**Affiliations:** 1 Department of Civil Engineering, University of Baghdad, Baghdad 17001, Iraq; e.mahmood1901p@coeng.uobaghdad.edu.iq (E.M.M.); a.allawi@uobaghdad.edu.iq (A.A.A.); 2 Department of Engineering and Technology, Texas A&M University-Commerce, Commerce, TX 75429, USA

**Keywords:** encased GFRP beam, high-strength concrete, strains, deformation, FE analysis, parametric study

## Abstract

There is an interesting potential for the use of GFRP-pultruded profiles in hybrid GFRP-concrete structural elements, either for new constructions or for the rehabilitation of existing structures. This paper provides experimental and numerical investigations on the flexural performance of reinforced concrete (RC) specimens composite with encased pultruded GFRP I-sections. Five simply supported composite beams were tested in this experimental program to investigate the static flexural behavior of encased GFRP beams with high-strength concrete. Besides, the effect of using shear studs to improve the composite interaction between the GFRP beam and concrete as well as the effect of web stiffeners of GFRP were explored. Encasing the GFRP beam with concrete enhanced the peak load by 58.3%. Using shear connectors, web stiffeners, and both improved the peak loads by 100.6%, 97.3%, and 130.8%, respectively. The GFRP beams improved ductility by 21.6% relative to the reference one without the GFRP beam. Moreover, the shear connectors, web stiffeners, and both improved ductility by 185.5%, 119.8%, and 128.4%, respectively, relative to the encased reference beam. Furthermore, a non-linear Finite Element (FE) model was developed and validated by the experimental results to conduct a parametric study to investigate the effect of the concrete compressive strength and tensile strength of the GFRP beam. The developed FE model provided good agreement with the experimental results regarding deformations and damaged patterns.

## 1. Introduction

Fiber Reinforced Polymer (FRP) materials are taking place in several applications in civil construction such as bridges and buildings, which are new or degraded structures [1]. There are different types of FRP composites, which include Carbon FRP (CFRP), Glass FRP (GFRP), and Basalt FRP (BFRP). The CFRP has more excellent mechanical properties and fatigue/creep/corrosion resistance. However, it is expensive, which limits some engineering applications [2]. The mechanical properties of GFRP and BFRP are good with the desirable material cost. However, their long-term properties under the concrete alkaline environment are relatively poor due to some possible chemical degradation reactions [3]. The advantages of using GFRP pultruded beams are high strength and stiffness, lightweight, free formability, high durability even under offensive environments, low thermal conductivity, and corrosion resistance [1,4]. Moreover, encasing these beams in concrete could improve their long-term durability. On the other hand, the application of high-strength concrete has increased in construction, with the rapid progress of concrete technology [5,6,7,8,9]. However, the material behavior of high-strength concrete is different from normal strength concrete. For instance, the modulus of elasticity, tensile, and shear strength of concrete don’t increase in direct proportion to the compressive strength [10]. The greater concern is the higher brittleness of high-strength concrete compared to that of normal strength concrete. Therefore, the advantages of encased pultruded GFRP section with concrete are significantly reducing the deformation and weight of the structure, enhancing ductility, increasing the flexural stiffness and strength capacity of a structure, and preventing buckling of the GFRP section [11,12]. At the same time, the concrete around the GFRP section improves the fire strength of the GFRP section. Therefore, this kind of beam is used in many civil engineering structures and infrastructures like bridges, marine structures, and buildings [11].

Based on the literature, understanding the behavior of the GFRP-section with RC was the main objective of many researchers [13,14,15,16]. The first research on GFRP material was studied by Boller [13] to evaluate the stress and rupture of the GFRP laminates, which were tested in flexure, tension, and shear. The laminate containing the straight fiber was the strongest. Ascione et al. [14] presented experimental results of the mechanical performance of composite beams obtained by bonding GFRP rectangular panels using an epoxy structural adhesive to form an I-section beam. The flexural response of these bonded beams was compared with those obtained by the pultrusion process with the same geometrical and material properties. No significant loss of performance emerged in terms of failure load. Moreover, an increase in pre-failure stiffness was observed. Youssef [11] studied the flexural and shear behavior of concrete members reinforced with GFRP rebars and embedded with pultruded GFRP structural sections. Moreover, an analytical model was developed to determine the axial load-bending moment interaction diagrams of the experimentally tested specimens. The results showed that the encased pultruded GFRP specimens obtained higher capacity and lower ductility relative to the specimens with steel or GFRP rebars. Yuan and Hadi [15] studied the bond behavior of the GFRP I-section encased in concrete by using a push-out test. The longer bond length and sand coating improved the ultimate bond stress. Hadi and Yuan [16] investigated pultruded GFRP I-section encased in concrete reinforced by steel or GFRP rebars. The tested beams exhibited ductile response and higher ultimate load than the reference beam, and the ductility, stiffness, and strength were affected by the type of tensile reinforcement while less affected by the location of pultruded beams.

Previous numerical investigations were conducted on the behavior of encased GFRP beams under the effect of static and impact loading [17,18,19,20]. The encased pultruded GFRP, concrete damaged plasticity, and failure progression of the GFRP profile based on the Hashin damage model were implemented in these models [17]. The peak loads were enhanced by increasing the composite action between the GFRP beam and concrete by using shear connectors. Moreover, improvements in the peak loads were obtained as the concrete compressive strength increased. The encased GFRP I-beam with links and studs enhanced the ductility and using bar chip fiber in mixing concrete enhanced the first crack load of the composite beam [19]. The matrix crack, local delamination, and concrete splitting and pullout were the common modes of failure of the composite beams [20].

Previous studies were reported on the behavior of normal- and high-strength concrete reinforced with GFRP rebars [21,22,23]. Almusallam [21] studied the behavior of concrete cylinders with normal- and high-strength concrete with GFRP jackets, which increased both the compressive strength and ductility of normal concrete. The effect of confinement was substantial for normal-strength concrete and marginal for high-strength concrete. El-nemr et al. [22] investigated the flexural performance of GFRP bars-reinforced normal- and high-strength concrete beams. The stiffness of both concretes was reduced after cracking and then showed similar performance until failure. The post cracking of normal-strength concrete was lower than high-strength concrete beams when the same reinforcement stiffness was provided. Saleh et al. [23] analyzed the bond behavior of GFRP in high-strength concrete. The reduction rate in bond strength decreased with increasing the rebar size.

Currently, research on encased GFRP beams with high-strength concrete is very limited. This manuscript adds valuable test data for encased pultruded GFRP I-beam with high-strength concrete under static loading. From this source and the latest studies in this field, this paper provides experimental and numerical investigations on the flexural performance of RC specimens composite with encased pultruded GFRP I-sections. Five simply supported composite beams were tested. Besides, the effect of using shear studs to improve the composite interaction between the GFRP beam and concrete as well as the effect of web stiffeners were explored. In addition, a non-linear FE model was developed and validated with the experimental results to conduct a parametric study to investigate the effect of the concrete compressive strength and tensile strength of the GFRP beam.

## 2. Experimental Program

Five simply supported composite beams were tested to investigate the static flexural behavior of encased GFRP pultruded I-beams with high-strength concrete. The test matrix for the conducted experimental program is listed in Table 1. In the adopted nomenclature, the symbol Ref refers to the reference beam, which was an RC beam and tested for comparison purposes. The symbol EG refers to the encased GFRP composite beam. The subsequent symbols S and W indicate using shear connectors and web stiffener, respectively.

### 2.1. Details of the Tested Specimens

**Figure 1:**
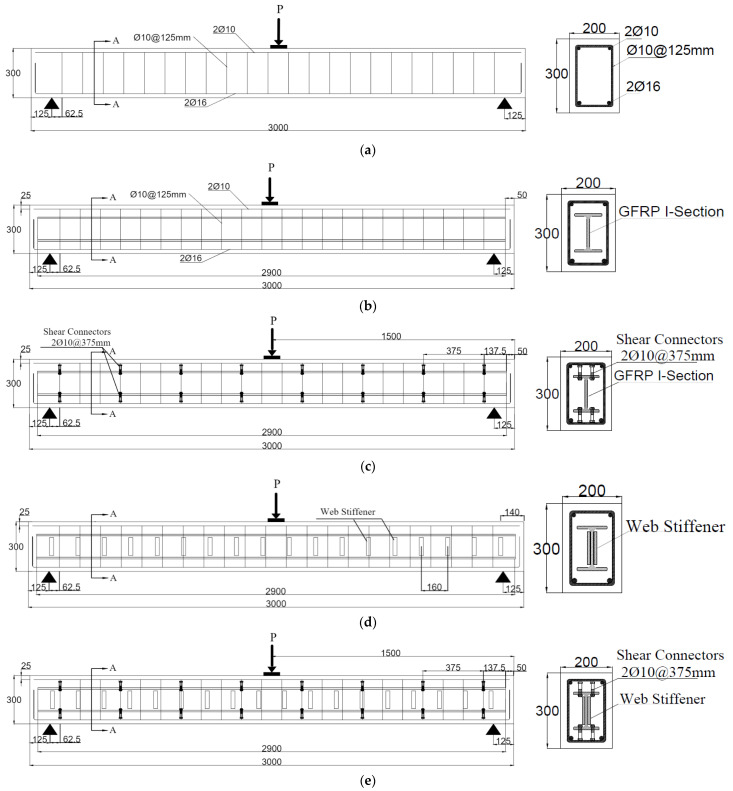
Details of the tested specimens. (All dimensions are in mm). (**a**) Ref beam. (**b**) EG beam. (**c**) EGS beam. (**d**) EGW beam. (**e**) EGSW beam.

**Figure 2:**
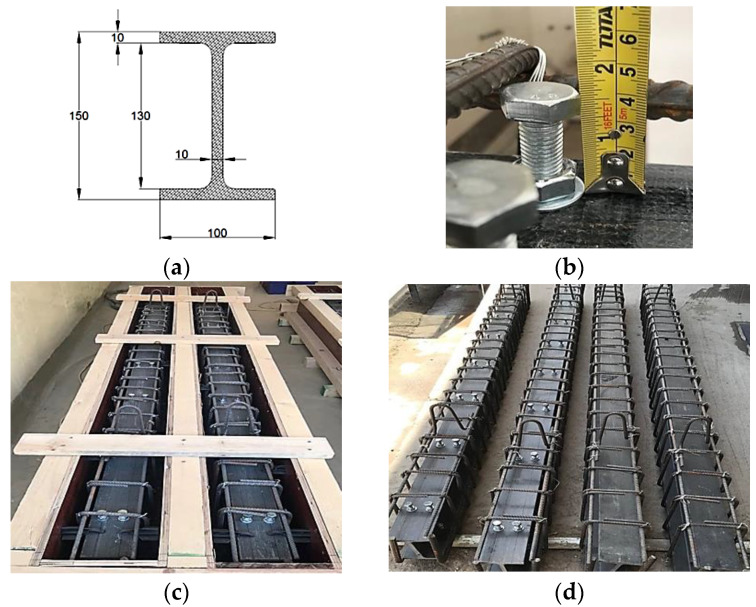
Details of the GFRP cross-section dimensions, studs, and reinforcements. (All dimensions are in mm). (**a**) Cross-section of GFRP I-beam. (**b**) Set of the shear connector. (**c**) GFRP I-beams with reinforcement. (**d**) GFRP I-beams with reinforcement and shear.

**Figure 3:**
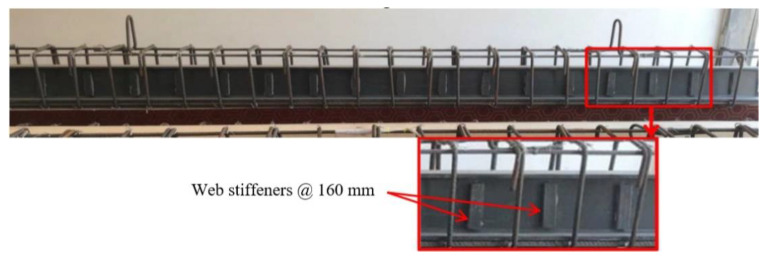
GFRP I-beams with reinforcement and web stiffener.

All beams were simply supported with overall and effective lengths of 3000 mm and 2750 mm, respectively. Rectangular cross-sections with 200 mm in width and 300 mm in height were adopted. The details of the tested beams are shown in Figure 1. The tested beams were reinforced in the tension zone by 2Ø16 mm and in the compression zone by 2Ø10 mm. The transverse reinforcement consisted of closed stirrups of 10 mm diameter at an equal spacing of 125 mm. These steel reinforcements were designed according to ACI 318–19. For the encased beams (EG, EGS, EGW, and EGSW), an identical GFRP-section of 150 mm depth (d_G_), 100 mm flange width (b_f_), and flange and web thickness of 10 mm (t), as shown in Figure 2a. The encased GFRP section was positioned at the center of the concrete cross-section of each tested beam. To increase the composite action between the encased GFRP section and concrete, steel shear connectors with a diameter of 12 mm and a height of 60 mm were used. The shear connectors were fabricated at the top flange of the GFRP section using hexagonal nuts with a diameter of 18 mm, as shown in Figure 2b. These connectors were arranged in two rows at a longitudinal spacing of 375 mm, as illustrated in Figure 2c,d. Rectangular prisms with dimensions of 110 mm × 25 mm × 10 mm were prepared and attached to the GFRP I-beams on both sides, as web stiffeners, at a longitudinal spacing of 160 mm to strengthen the web against undesired premature failures, as shown in Figure 3.

### 2.2. Material Properties

The concrete mix proportion of the used high-strength concrete is listed in Table 2. The maximum aggregate size was 12 mm. The compressive strength and modulus of elasticity of 53.8 MPa and 31,000 MPa, respectively, were obtained from testing concrete cylinders with 150 × 300 mm dimensions according to ASTM C39-39M [24] and ASTM C469-469M [25], respectively.

Tension tests were carried out on steel rebars of 16 and 10 mm diameters. Three specimens for each diameter were tested in the Consulting Engineering Bureau, College of Engineering, the University of Baghdad according to ASTM A615/A615M-18 [26]. The obtained yield stresses were 520 and 408 MP, respectively. Whereas the ultimate stresses were 687 and 466 MPa, respectively.

The GFRP I-beams used in this study were made by an international manufacturer of FRP products (Dura composites, United King). They were made of isophthalic polyester resins reinforced with E-glass fibers. The mechanical and geometric properties of the pultruded GFRP I-section are listed in Table 3. These properties were obtained from standard tests according to ASTM D695–15 [27] and ISO 527-4:2021 [28] for the compressive and tensile properties, respectively.

### 2.3. Experimental Setup and Instrumentations

**Figure 4:**
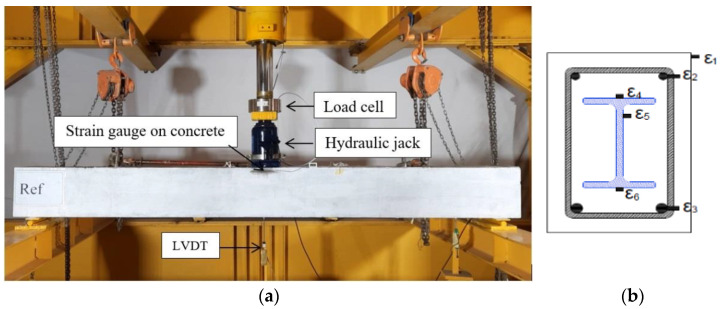
Experimental setup and instrumentations. (**a**) Test set-up. (**b**) Strain gauges.

The specimens were tested as simply supported beams under the effect of a concentrated load at the mid-spans. A hydraulic jack, with 490 kN capacity, was used to apply the load. The applied load and corresponding deflection were recorded using a load cell and linear variable differential transforms (LVDT), respectively, as shown in Figure 4a. Electrical strain gauges (*ε*_1_ to *ε*_6_) were used to measure the strains in the steel rebars, concrete, and GFRP I-section at the mid-spans, as illustrated in Figure 4b.

## 3. Experimental Results and Discussion

### 3.1. Load-Deflection Curves

**Figure 5:**
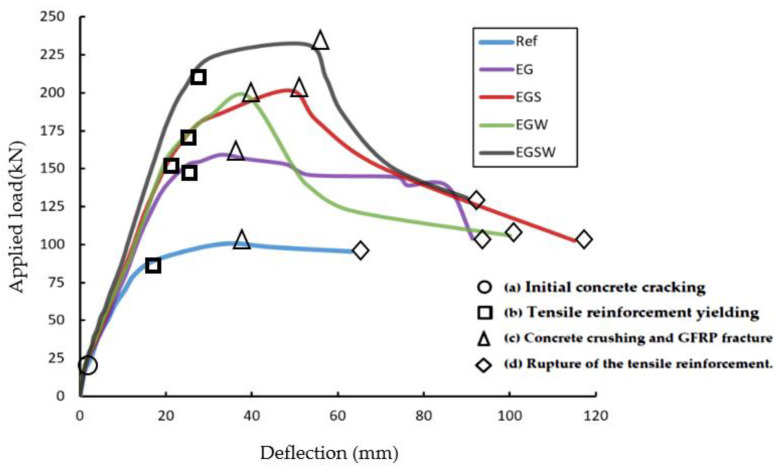
Load versus deflection relationships for the tested beams.

The load versus mid-span deflection curves for the tested beams are shown in Figure 5. The relationships show four distinguished stages: (a) initial concrete cracking, (b) tensile reinforcement yielding, (c) concrete crushing and fracture of the GFRP beams, and (d) rupture of the tensile reinforcement. All curves started with the same abrupt slop in the first stage until the loading level of 15 kN. Then, the curves started to deviate from each other. As the applied load increased, the slops of the curves of specimens with shear connectors and web stiffeners became steeper towards the ultimate points. The peak loads of the tested beams EG, EGS, EGW, and EGSW were 58.3%, 100.6%, 97.3%, and 130.8% higher than the reference beam Ref, respectively, as listed in Table 4. After reaching the ultimate points, the applied loads were dropped due to the fracture of the GFRP beams. The bond-slip was responsible for the reduction in the slope beyond the ultimate load. Compared to the encased GFRP beams with shear connectors or web stiffeners or both (EGS, EGW, and EGSW beams), the EG beam solely depended on the friction between the GFRP beam and concrete while the shear connectors and web stiffeners provided better anchorage and bond. Therefore, the shear connectors and web stiffeners were provided to treat the bond-slip problem, and it was proven in this research. The ultimate load and corresponding central displacement are shown in Table 4. The central displacements were proportional to the ultimate load. More ductility was obtained when using the shear connectors in specimens EGS and EGSW.

### 3.2. Crack Patterns and Failure Modes

**Figure 6:**
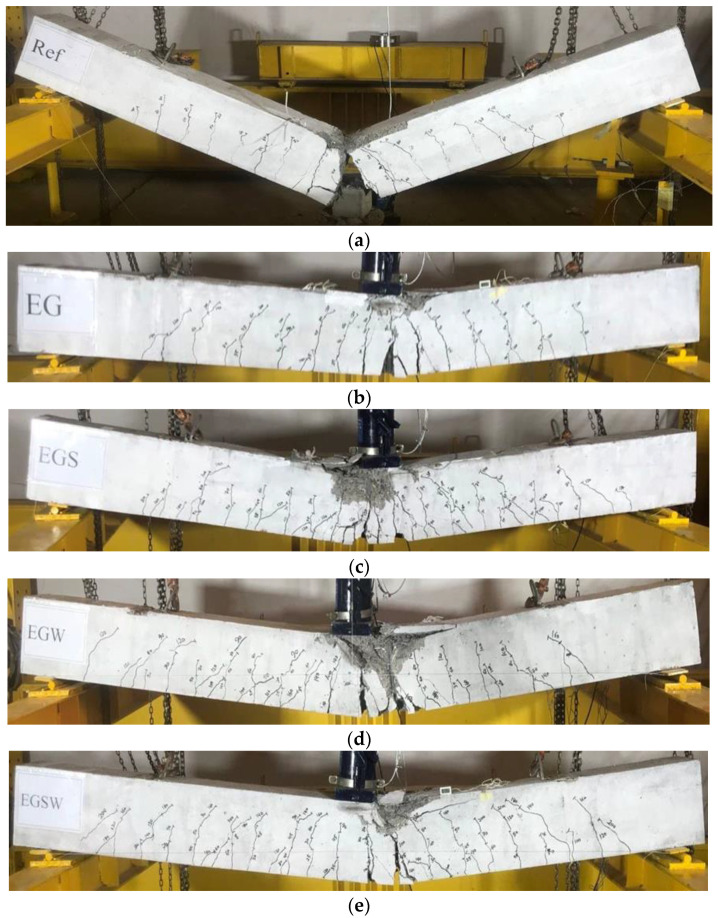
Failure modes of the tested beams. (**a**) Beam Ref. (**b**) Beam EG. (**c**) Beam EGS. (**d**) Beam EGW. (**e**) Beam EGSW.

The initial crack load for each specimen is listed in Table 4. Cracks were initiated when the tensile stress at the bottom fiber of the tested beam was larger than the tensile strength of concrete. Then, the stresses started to be transferred to the steel rebars. The initial crack loads were very close to each other because the encased GFRP beam didn’t contribute to the first stage of loading. As the applied load increased, new cracks were formed and appeared. The crack patterns and failure modes are shown in Figure 6. The reference beam, Ref, experienced yielding of steel reinforcement followed by crushing of concrete in the compression zone and more cracks were developed along with the depth of the concrete cross-section. Finally, a rupture in the bottom steel rebars occurred. This rupture caused the cracks to penetrate the specimen’s cross-section and led to cutting the beam into two separate parts, as shown in Figure 6a. For the encased beams EG, EGS, EGW, and EGSW, the major cracks were developed at the mid-spans and propagated towards the compression zones. After reaching the ultimate loads, major cracks were observed joining each other and shear cracks were created. The final failure modes were concrete crushing followed by cover spalling and buckling of the top steel rebars. At the same time, ruptures in the GFRP beams and the bottom steel rebars occurred. As shown in Figure 6c–e, the shear connectors and web stiffeners increased the tested beams’ rigidity, which caused more flexural cracks formation in these beams. Moreover, the crushed concrete zones (region of the compression zone) were deeper and the crack spacing in the tension zone was narrower.

### 3.3. Load-Strain Relationships

**Figure 7:**
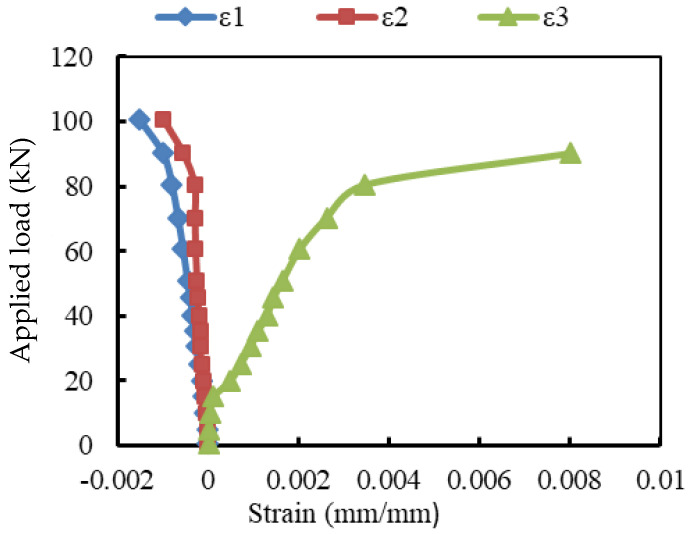
Load- strain relationship at mid-span of beam Ref.

**Figure 8:**
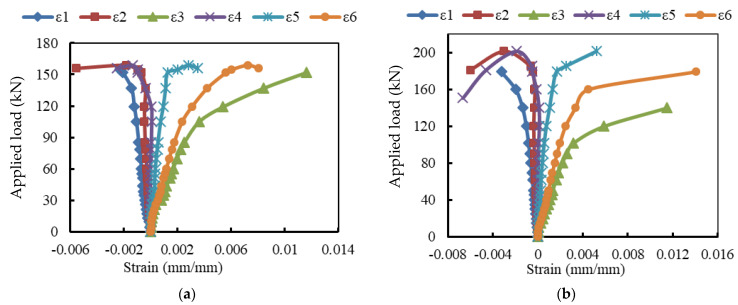

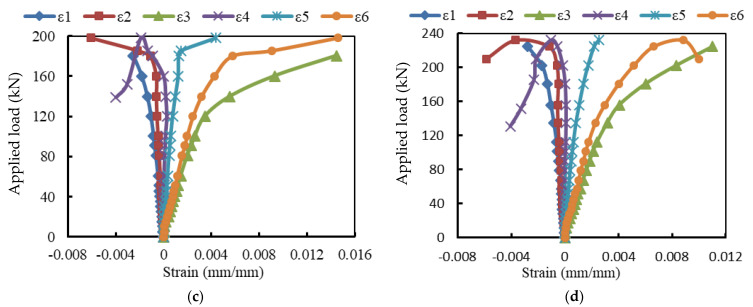
Load-strain relationships at mid-span of the encased beams. (**a**) Beam EG. (**b**) Beam EGS. (**c**) Beam EGW. (**d**) Beam EGSW.

**Figure 9:**
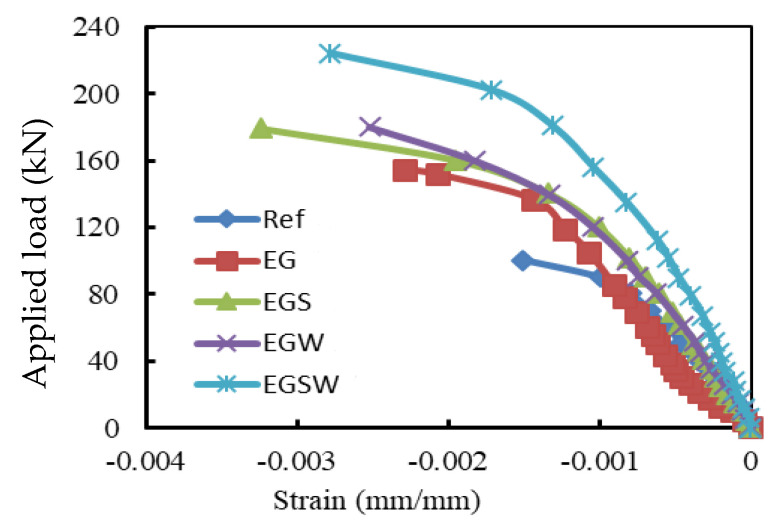
Load-strain relationships of concrete in compression zone at mid-span.

The strain records are illustrated in Figure 7, Figure 8 and Figure 9. For the reference beam Ref, the maximum measured compressive strain in concrete was 0.0015 when yielding in the tensile reinforcement occurred. Whereas these values were 0.0023, 0.0032, 0.0025, and 0.0028 for the encased beams EG, EGS, EGW, and EGSW, respectively. The GFRP beam enhanced the encased beams’ capacities and led to increases in the measured ultimate strains in concrete (see Figure 9). Based on these records, the tensile reinforcements in all beams firstly reached yielding at different loading levels. The yielding loads for the encased beams EG, EGS, EGW, and EGSW were 99.1 kN, 101.1 kN, 120.5 kN, and 155.3 kN with 23.7%, 26.2%, 50.1%, and 93.7% increases relative to the reference beam, respectively. Table 5 lists the maximum strain measurements in concrete and steel reinforcement. The measured strains in the tensile and compressive steel reinforcement for the encased beams were higher than those of the reference beam. The EGW beam experienced the highest tensile strain of 0.0145. The existence of studs and web stiffeners in beam EGSW improved the beam rigidity and released the tensile strains in the steel reinforcement. Therefore, this beam showed much improvement in the flexural behavior relative to the other encased beams. The strains in the GFRP beams increased almost linearly until failure. From these values of strain measurements in each part of the GFRP beams (top flange, bottom flange, and web), the GFRP beams contributed to providing more strength to the encased beams. The contribution of the GFRP beams increased by adding the studs and web stiffeners because the bottom flanges of these beams exhibited additional strains due to increasing the composite interaction with concrete. 

### 3.4. Ductility

**Figure 10:**
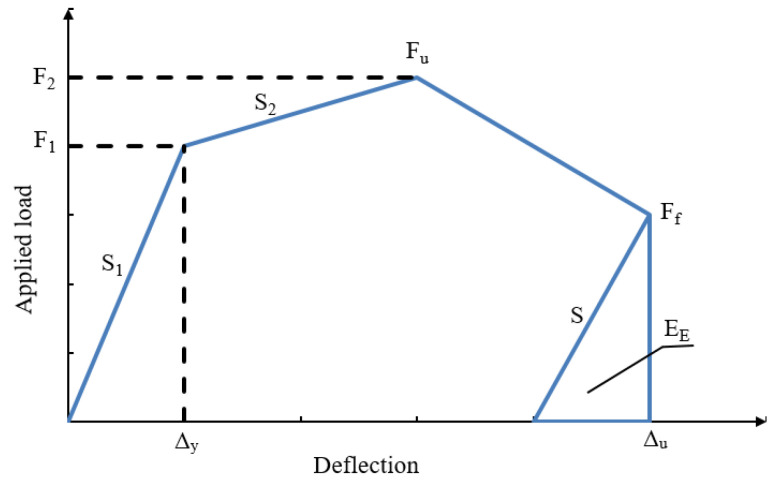
The ductility mode that used in this study [29].

There are many definitions for ductility and ductility index of conventional RC structures with only steel rebars. The ratio of deflection at ultimate load to the deflection at yield load can define the ductility of these members. The FRP materials have a linear stress-strain relationship until failure, and the energy released for FRP beams is linear, which is different from that for steel reinforcement. Therefore, the ductility in this study is based on the energy theory [29]. The ductility (*μ_E_*) was calculated based on Equation (1) depending on the load-deflection relationships for the tested beams.

(1) $$\mu_{E\;}=\frac{1}{2}\;\left(\frac{E_{T}}{E_{E}}+1\right)$$

where *E_T_* is the total energy calculated from the area under the load-deflection curve, *E_E_* is the stored elastic energy calculated from the load-deflection relationships as shown in Figure 10. The slope (*S*) was obtained from the following equation:

(2) $$S=\;\left(\frac{P_{1\;}S_{1}+\left(P_{2}-P_{1}\right)S_{2}}{P_{2}}\right)$$

where $P_{1\;}$ was the load at the end of the elastic stage, $S_{1}\;$ was the slop of the elastic stage, $P_{2\;}$ was the peak load at the end of the second line, and $S_{2}$ was the slope of the second line.

The total energy, elastic energy, and ductility of the tested specimens are illustrated in Table 6. The ductility of beam Ref was 3.52, which was smaller than the encased beams. The GFRP beams improved the ductility by 21.6% relative to the reference one. Moreover, the shear connectors, web stiffeners, and both improved the ductility by 185.5%, 119.8%, and 128.4%, respectively, relative to the encased beam EG.

## 4. Numerical Modeling

Finite Element (FE) models were developed, using the general-purpose FE code Abaqus [30], to simulate the three-dimensional (3D) modeling of encased pultruded GFRP beams under static loading.

### 4.1. FE Modelling of Encased Beam

**Figure 11:**
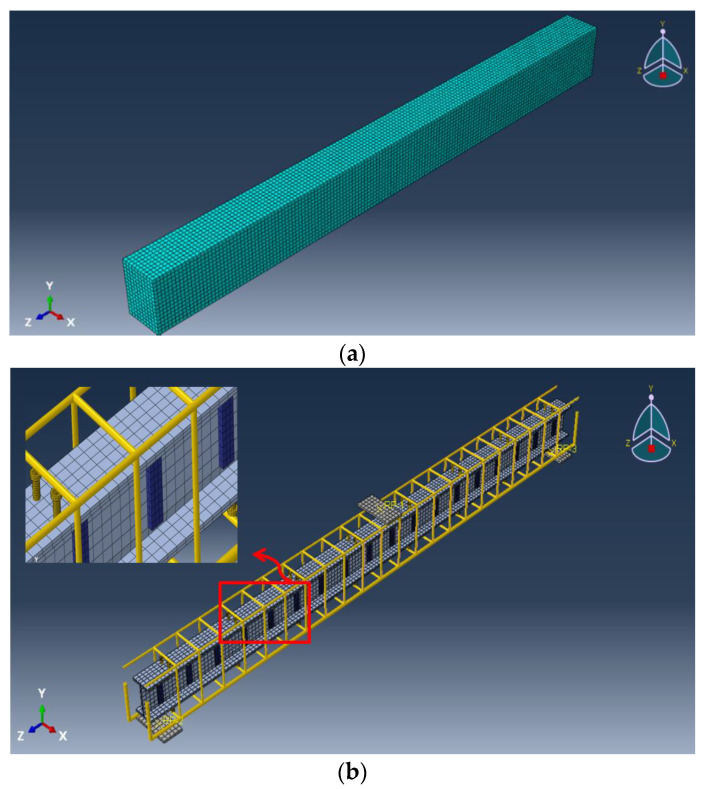
FE mesh shows the different components of the encased beams. (**a**) Mesh of the concrete beam. (**b**) Mesh of the studs, reinforcements, and GFRP beam.

Different element types were used to model the different components of the encased beams (concrete, steel reinforcement, pultruded GFRP beam, shear connectors, and web stiffeners). Continuum eight-node solid element with reduced integration (C3D8R) was used to simulate the nonlinear behavior of concrete, shear connectors, and steel plates. The interface between the bearing plates and concrete was modeled as a surface-to-surface interaction. The pultruded GFRP beam and web stiffeners were modeled by using the four-node doubly curve shell element with reduced integration (S4R). The longitudinal and transverse reinforcements were modeled using the 2-node linear 3D truss element (T3D2). Several nonlinear analyses with different element sizes were performed to select the best mesh size to obtain accurate results at a reasonable solving time. The FE mesh showing the different components of the encased beams is illustrated in Figure 11.

The experimental boundary conditions were adopted in the FE analysis as simply supported beams. The first support was constrained in the Y- and Z-directions, representing the hinge support. At the same time, the second support was constrained only in the Y-direction, which expressed the roller support. The whole model was constrained in the X-direction. The full bond technique was assumed to simulate the connection between the concrete and steel rebars. However, the bond between the GFRP beam surface and the surrounded concrete was simulated using surface-to-surface contact pairs. The contact property was represented by the tangential behavior with a penalty friction formulation. The tangential shear stress was adopted from the push-out test as 0.422 MPa [31] and the friction coefficient was used equally at 0.55 according to the test of Hadi and Yuan [16]. However, the full bond between the shear studs and concrete was assumed. The displacement-controlled strategy was employed to load the analyzed beams by defining the vertical displacement value of the reference point.

### 4.2. Material Modeling

**Figure 12:**
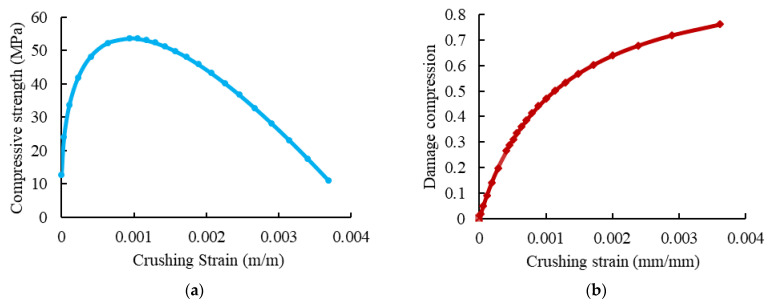
The behavior of concrete under compression. (**a**) Concrete compressive stress-strain curve. (**b**) Compressive damage evolution of concrete.

**Figure 13:**
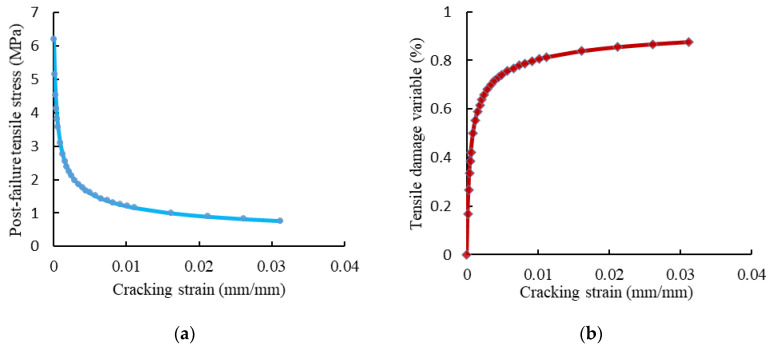
The behavior of concrete under tension. (**a**) Post-failure behavior of concrete. (**b**) Tensile damage evolution of concrete.

The compressive behavior of concrete was defined using the concrete damaged–plasticity (CDP) model [32]. In this model, the material principal failure mechanisms were compressive crushing, tensile cracking, and loss of elastic stiffness. The value of the dilation angle *ψ* was 36°, the value of the plastic flow potential eccentricity (e) was 0.1, and the ratio of the initial equibiaxial compressive yield stress to initial uniaxial compressive yield stress (σ_bo_/σ_co_) was 1.16. Moreover, the coefficient (K_c_) was 2/3 and the viscosity parameter was 0.001. The elastic properties of concrete were specified according to the ACI 363R-92 [5] and ACI 318-19 [33] guidelines. The used uniaxial compressive stress-strain and damage compression–crushing strain relationships of concrete are shown in Figure 12. The concrete behavior under uniaxial tension was represented by the tension softening mechanism and tension stiffening due to the tensile resistance of concrete surrounding the tensile reinforcement, which was forced by bond stresses to extend simultaneously with reinforcement [34,35]. Figure 13 presents the adopted post-failure tensile stress and tensile damage with cracking strains curves.

The steel reinforcement was simulated as an elastic–perfectly plastic material. The modulus of elasticity and yielding stress were used to define the stress-strain curve. The degradation in the GFRP I-beam was modeled according to Hashin’s criteria [36]. According to the damage evolution law, the material stiffness of the GFRP I-beam degraded after satisfying the damage initiation criteria. The Hashin damage initiation criterion is used in Abaqus, along with a progressive damage variable based on stress state and fracture energy G_f_ as listed in Table 3 and Table 7, respectively.

### 4.3. Validations of the FE Results

**Figure 14:**
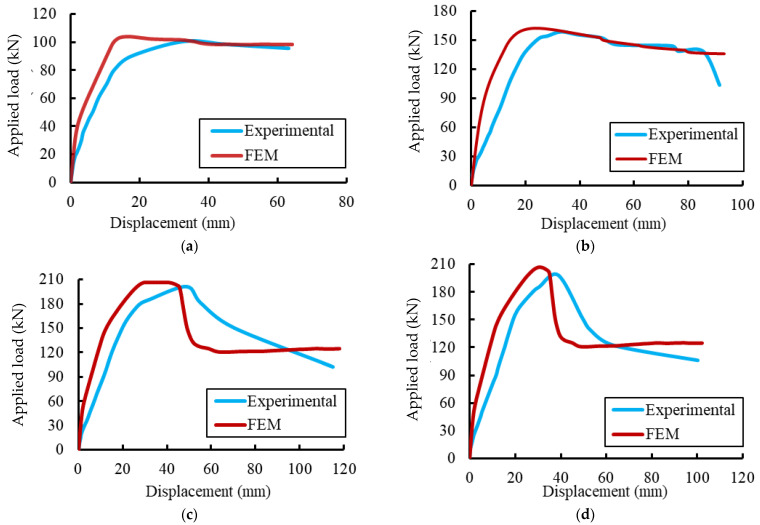

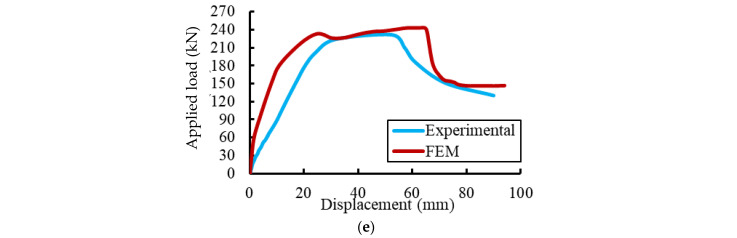
Comparison between FE and experimental deformations. (**a**) Specimen Ref. (**b**) Specimen EG. (**c**) Specimen EGS. (**d**) Specimen EGW. (**e**) Specimen EGSW.

**Figure 15:**
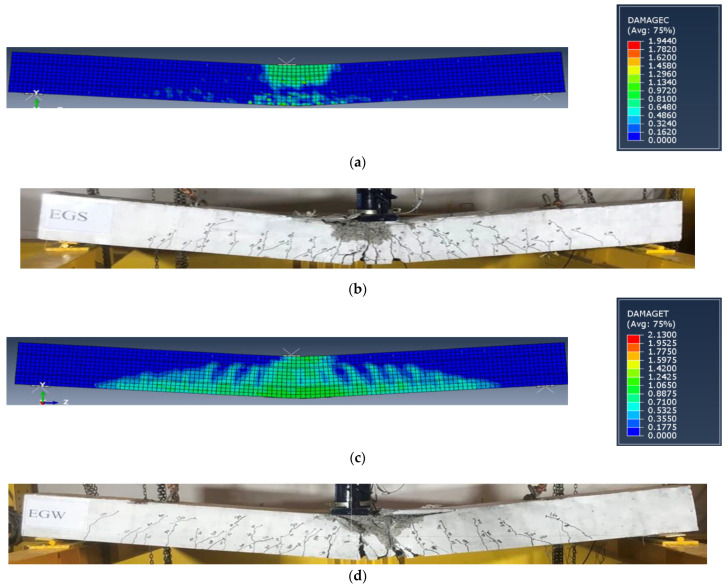
Cracking pattern comparisons. (**a**) FE crack pattern for specimen EGS. (**b**) Experimental crack pattern for specimen EGS. (**c**) FE crack pattern for specimen EGW. (**d**) Experimental crack pattern for specimen EGW.

The conventional load-deformation curves for the tested specimens were used to validate the FE results, as shown in Figure 14 and Figure 15. Initially, the FE deformations exhibited linear elastic behaviors with a higher stiffness than the experimental records. This difference in behavior could be attributed to the used constitutive models for materials and the full bond assumed between the concrete and steel rebars in the FE analysis. As the applied load gradually increased, cracks were formed and the non-linear deformation behaviors were obtained in good agreement with the experimental results. Table 8 summarizes the experimental and FE results regarding the maximum deflections and loads. The comparisons show that the difference between the maximum applied loads reached about 4.25% for the specimen EGW.

The FE crack patterns for concrete were represented in Abaqus by defining the post-cracking damage properties for the CDP model by visualizing the tensile damage at the integration point DAMAGET. Figure 15 shows a closed agreement between the FE and experimental crack patterns, indicating that the proposed FE model effectively predicted the failure behavior of the tested specimens. Based on these comparisons, the proposed FE model can simulate the tested specimens with and without the GFRP beam. Therefore, the proposed model was used to evaluate a comparative parametric study.

## 5. Parametric Study

The influences of the compressive strength of concrete and tensile strength of the GFRP beam on the flexural behavior of encased GFRP I-beam were investigated using the verified FE model.

### 5.1. Effect of the Concrete Compressive Strength

**Figure 16:**
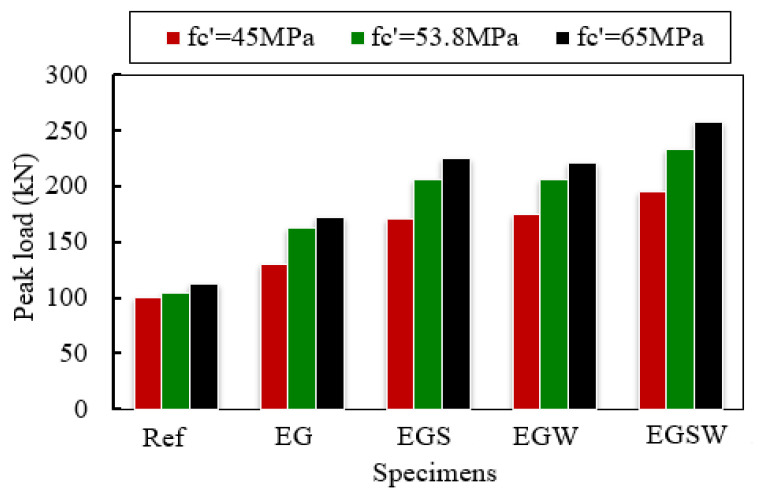
The variation of ultimate load as compressive strength changes.

The influence of various concrete compressive strengths of 45 MPa, 53.8 MPa, and 65 MPa on the flexural performance of encased beams under static load was studied. The different compressive strengths were implemented in Abaqus through the stress-strain curves as well as the CDP model. The peak loads of the analyzed specimens are shown in Figure 16. Enhancements in the peak loads, service load, and maximum deflections of the analyzed specimens were obtained as the concrete compressive strength increased, as listed in Table 9. The percentages were estimated for the reference concrete compressive strength of 45 MPa. The peak loads increased with increasing the compressive strength of concrete. For the reference beam Ref, the peak load increased by 3.76% and 11.92% for the compressive strength of 53.8 MPa and 65 MPa, respectively, relative to the compressive strength of 45 MPa. The most enhancements in the peak loads were for the encased beam EG, which were 24.78% and 32.32% for compressive strengths of 53.8 MPa 65 MPa, respectively, concerning the compressive strength of 45 MPa. Moreover, the mid-span deflections at the peak loads were reduced as the concrete compressive strength increased.

### 5.2. Effect of the Tensile Strength of the GFRP Beam

**Figure 17:**
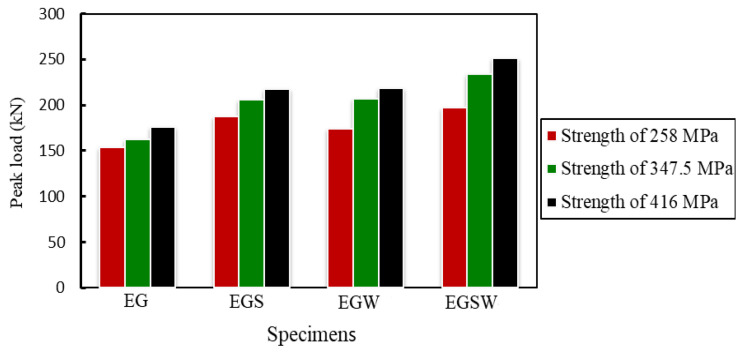
The variation of the peak is loads based on the GFRP tensile strength.

To study the effect of tensile strength of GFRP I-beam on the flexural behavior of the encased beams, two tensile strengths of 258 MPa and 416.6 MPa [37] were investigated, besides the tensile strength of 347.5 MPa, which was obtained in this study according to (ASTM Designation: D 695–15) [27]. The peak loads and the corresponding mid-span deflections increased as the tensile strength of GFRP increased as listed in Table 9 and shown in Figure 17. In these comparisons, the reference tensile strength was 258 MPa. The increase in the peak loads ranged between 6% and 18% when the tensile strength was 347.5 MPa, while the improvements ranged between 14% and 27% for tensile strength of 416 MPa, as listed in Table 10.

## 6. Conclusions

This paper provides experimental and numerical investigations on the flexural performance of RC specimens composite with encased pultruded GFRP I-sections. Five simply supported composite beams were tested in this experimental program to investigate the static flexural behavior of encased GFRP pultruded I-beams with high-strength concrete. Besides, the effect of using shear studs to improve the composite interaction between the GFRP beam and concrete as well as the effect of web stiffeners of GFRP were explored. Moreover, a non-linear FE model was developed and validated by the experimental results to conduct a parametric study to investigate the effect of the concrete compressive strength and tensile strength of the GFRP beam. The following conclusions can be drawn based on the experimental and FE results.

1. Encasing the GFRP beam with concrete enhanced the peak load by 58.3%. Using shear connectors, web stiffeners, and both improved the peak loads by 100.6%, 97.3%, and 130.8%, respectively, relative to the classical reinforced concrete. The shear connectors and web stiffeners increased the beams’ rigidity. In addition, the GFRP beams improved the ductility by 21.6% relative to the reference one. Moreover, the shear connectors, web stiffeners, and both improved the ductility by 185.5%, 119.8%, and 128.4%, respectively, relative to the reference beam.
2. The strains of the pultruded GFRP beams increased almost linearly until failure. The GFRP beams contributed to providing more strength to the encased beams. The contribution of the GFRP beams increased by adding the studs and web stiffeners because the bottom flanges of these beams exhibited additional strains due to increasing the composite interaction with concrete.
3. The peak loads increased with increasing the compressive strength of concrete. For the reference beam without a GFRP beam, the peak load increased by 3.76% and 11.92% for the compressive strength of 53.8 MPa and 65 MPa, respectively. However, the most enhancements in the peak loads were for the encased beam, which were 24.78% and 32.32% for the compressive strengths of 53.8 MPa 65 MPa, respectively, with respect to the compressive strength of 45 MPa.
4. The peak loads and the corresponding mid-span deflections increased as the tensile strength of GFRP increased. The increase in the peak loads ranged between 6% and 18% when the tensile strength was 347.5 MPa, while the improvements ranged between 14% and 27% for the tensile strength of 416 MPa.

## Figures and Tables

**Table 1:** The test matrix for the conducted experimental program.

Specimen Encoding	GFRP I-Section	Shear Connectors	Web Stiffeners
Ref	-	-	-
EG	√	-	-
EGS	√	√	-
EGW	√	-	√
EGSW	√	√	√

√ means yes.

**Table 2:** Concrete mix proportion.

	Cement (kg/m^3^)	Fine Aggregate (kg/m^3^)	Coarse Aggregate (kg/m^3^)	Water (kg/m^3^)	Admixture (kg/m^3^)
Amount	475	880	910	165	15.25

**Table 3:** Mechanical and geometrical properties of the GFRP I-beam.

Mechanical Properties	Value (MPa)	Geometrical Properties	Value
Transverse Compressive Strength	118.3	Area	3300 mm^2^
Longitudinal Compressive Strength	326.14	Perimeter	680 mm
Longitudinal Tensile Strength	347.5	Moment of inertia	11,647,500 mm^4^
Longitudinal Modulus of elasticity	27,100	Mass	5.94 kg/m
Transverse Modules of elasticity	6800	Web and flange thickness	10 mm

**Table 4:** Comparisons of the initial crack load, ultimate load, and central displacement.

Specimens	Initial Crack Load (kN)	% Change	Ultimate Load (kN)	% Change	Central Displacement (mm)	% Change
Ref	19.93	-	100.46	-	32.80	-
EG	20.24	+1.5	159.04	+58.3	33.07	+0.8
EGS	19.73	−1.0	201.54	+100.6	48.68	+48.4
EGW	20.12	0.9	198.24	+97.3	38.96	+18.8
EGSW	22.26	+11.7	231.88	+130.8	52.56	+60.2

**Table 5:** Maximum strain measurements in concrete and steel reinforcement.

Specimens	Strain in Concrete ε_1_ (mm/mm)	Change (%)	Strain in Compression Reinforcement ε_2_ (mm/mm)	Change (%)	Strain in Tensile Reinforcement ε_3_ (mm/mm)	Change (%)
Ref	0.0015	-	0.001	-	0.008	-
EG	0.0023	53	0.0055	450	0.0116	45
EGS	0.0032	116	0.0065	550	0.0115	44
EGW	0.0025	68	0.00615	515	0.0145	81
EGSW	0.0028	85	0.00588	488	0.011	38

**Table 6:** The ductility of the tested specimens.

Specimen	Slope *S*_1_	Slope *S*_2_	Slope *S*	Total Energy E_T_ (kN·mm)	Elastic Energy E_E_ (kN·mm)	Ductility *μ_E_*	% Change
Ref	6.1	0.9	5.1	5443	900	3.52	-
EG	6.3	0.9	6.1	11,933	1576	4.28	21.6
EGS	6.8	1	6.1	16,344	852	10.05	185.5
EGW	6.7	1.6	6.3	12,962	895	7.74	119.8
EGSW	7.6	0.4	7.4	17,397	1154	8.04	128.4

**Table 7:** Fracture energy of pultruded GFRP I- section beam.

Mechanical Properties Data	Value (N/mm)
Longitudinal tensile fracture energy	4.76
Longitudinal compressive fracture energy	0.375
Transverse tensile fracture energy	5
Transverse compressive fracture energy	0.55

**Table 8:** Comparison between the FE and experimental results.

Beam	Exp. Results		FE Results		Change (%)	
	Ultimate Load P_u_ (kN)	Max. Disp. (mm)	Ultimate Load P_u_ (kN)	Max. Disp. (mm)	Ultimate Load	Max. Disp.
Ref	100.46	63	104.24	64.11	3.7	1.76
EG	159.04	91	162.51	93.19	2.18	2.4
EGS	201.55	115	206.02	120.12	2.22	4.45
EGW	198.24	100	206.67	102.24	4.25	2.24
EGSW	231.88	90	233.96	94.07	0.90	4.52

**Table 9:** Effect of the Concrete Compressive Strength.

Beams	Compressive Strength (MPa)	Peak Load Pu (kN)	Deflection at Peak Load (mm)	Increase in Load (%)	Reduction in Deflection (%)
	45	100.46	18.32	-	-
Ref	53.8	104.24	16.56	3.76	9.61
	65	112.43	15.33	11.92	16.32
	45	130.23	26.38	-	-
EG	53.8	162.51	23.70	24.78	10.38
	65	172.32	18.41	32.32	43.29
	45	171.47	42.38	-	-
EGS	53.8	206.02	40.59	20.15	4.22
	65	225.03	31.05	31.24	26.73
	45	174.33	40.25	-	-
EGW	58.3	206.67	31.14	18.55	22.63
	65	221.27	27.39	26.93	31.95
	45	195.88	27.51	-	-
EGSW	53.8	233.96	25.37	19.44	7.78
	65	258.23	19.08	31.83	30.64

**Table 10:** Effect of the GFRP tensile strength.

Beam	Tensile Strength of GFRP (MPa)	Peak Load P_u_ (kN)	Deflection at Peak Load (mm)	Increase in Peak Load (%)	Increasing in Deflection (%)
	258	153.9	21.23	-	-
EG	347.5	162.51	23.70	5.59	11.63
	416	175.59	24.83	14.09	16.96
	258	187.43	28.38	-	-
EGS	347.5	206.02	40.59	9.92	43.02
	416	217.81	41.50	16.21	46.23
	258	174.05	27.97	-	-
EGW	347.5	206.67	31.14	18.74	11.33
	416	218.27	40.32	25.41	44.15
	258	197.33	19.75	-	-
EGSW	347.5	233.96	25.37	18.56	28.45
	416	251.43	26.40	27.42	33.67

## Data Availability

Not applicable.
